# The origin of the large $T_{\mathrm{c}}$ variation in FeSe thin films probed by dual-beam pulsed laser deposition

**DOI:** 10.1007/s44214-024-00058-0

**Published:** 2024-06-08

**Authors:** Zhongpei Feng, Hua Zhang, Jie Yuan, Xingyu Jiang, Xianxin Wu, Zhanyi Zhao, Qiuhao Xu, Valentin Stanev, Qinghua Zhang, Huaixin Yang, Lin Gu, Sheng Meng, Suming Weng, Qihong Chen, Ichiro Takeuchi, Kui Jin, Zhongxian Zhao

**Affiliations:** 1 Beijing National Laboratory for Condensed Matter Physics, Institute of Physics, Chinese Academy of Sciences, Beijing, 100190 China; 2 Songshan Lake Materials Laboratory, Dongguan, Guangdong 523808 China; 3 University of Chinese Academy of Sciences, Beijing, 100049 China; 4 Center for Intense Laser Application Technology, Shenzhen Technology University, Shenzhen, 518118 China; 5 Key Laboratory for Vacuum Physics, University of Chinese Academy of Sciences, Beijing, 100049 China; 6 CAS Key Laboratory of Theoretical Physics, Institute of Theoretical Physics, Chinese Academy of Sciences, Beijing, 100190 China; 7 Department of Materials Science and Engineering, University of Maryland, College Park, MD 20742 USA; 8 Maryland Quantum Materials Center, University of Maryland, College Park, MD 20742 USA; 9 Key Laboratory for Laser Plasmas (MoE), School of Physics and Astronomy, Shanghai Jiao Tong University, Shanghai, 200240 China; 10 Collaborative Innovation Center of IFSA, Shanghai Jiao Tong University, Shanghai, 200240 China

**Keywords:** High-temperature superconductivity, Iron chalcogenide superconductors, Pulsed laser deposition, High-throughput technique

## Abstract

FeSe is one of the most enigmatic superconductors. Among the family of iron-based compounds, it has the simplest chemical makeup and structure, and yet it displays superconducting transition temperature ($T_{\text{c}}$) spanning 0 to 15 K for thin films, while it is typically 8 K for single crystals. This large variation of $T_{\text{c}}$ within one family underscores a key challenge associated with understanding superconductivity in iron chalcogenides. Here, using a dual-beam pulsed laser deposition (PLD) approach, we have fabricated a unique lattice-constant gradient thin film of FeSe which has revealed a clear relationship between the atomic structure and the superconducting transition temperature for the first time. The dual-beam PLD that generates laser fluence gradient inside the plasma plume has resulted in a continuous variation in distribution of edge dislocations within a single film, and a precise correlation between the lattice constant and $T_{\text{c}}$ has been observed here, namely, $T_{\text{c}} \propto \sqrt{c- c_{0}}$, where *c* is the *c*-axis lattice constant (and $c_{0}$ is a constant). This explicit relation in conjunction with a theoretical investigation indicates that it is the shifting of the $d_{\text{xy}}$ orbital of Fe which plays a governing role in the interplay between nematicity and superconductivity in FeSe.

**Supplementary Information:**

The online version contains supplementary material available at 10.1007/s44214-024-00058-0.

The properties of iron-based superconductors are delicately controlled by subtle variation of their lattice parameters [1–4], and delineating the correlation between structure and the superconducting properties is an important path towards understanding the mechanism of superconductivity. Among the iron-based superconductor family, iron selenide (FeSe) has the simplest lattice structure and yet exhibits widely varying superconducting properties closely associated with minute changes in its lattice parameters [5–7]. A bulk FeSe single crystal becomes superconducting at a critical temperature $T_{\mathrm{c}} \approx 8\text{ K}$ [8], which can be boosted by modifying the lattice parameters: under high pressure, its $T_{\mathrm{c}}$ can be pushed up to ${\approx} 37\text{ K}$ with reduction in both the in-plane (*a*) and out-of-plane (*c*) lattice parameters and a decrease in the $c/a$ ratio [9–11]. By an alkali metal or organic molecule interlayer intercalation [12, 13], the *c*-axis lattice constant of a crystal can be increased, and concomitantly $T_{\mathrm{c}}$ is enhanced to 40–50 K. In our previous study, we have applied ionic gating on an FeSe film and raised its $T_{\mathrm{c}}$ from 10 to 45 K [14]. More strikingly, for a monolayer FeSe grown on a SrTiO_3_ substrate, the in-plane lattice expands by ${\approx} 3\%$ and the superconducting energy gap closes at $(\approx) 65\text{ K}$ [15], revealing an unusually large intrinsic tunability of superconductivity in FeSe.

Compared to the bulk and the monolayer limit, thin films of FeSe in the intermediate regime (with thicknesses ranging from several to hundreds of nanometers) provide an indispensable platform for performing fundamental studies and pursuing practical applications [16]. Pulsed laser deposition (PLD) has been a powerful and versatile technique for fabricating FeSe thin films [17]. Nevertheless, ubiquitous micron-scale particulates generated during the laser ablation process can induce complex variation in the local atomic structure of the films, which in turn can influence their physical properties [18]. For example, FeSe thin films grown by PLD on different substrates can result in different strain states displaying a range of transport properties spanning insulating to superconducting with $T_{\mathrm{c}}$ up to 15 K [19–24] which is nearly twice that of bulk FeSe, underscoring the importance of delicate microstructural variation influencing their superconducting properties. To date, however, a definitive description connecting superconductivity and the lattice parameters in FeSe has been lacking, hindering the understanding of superconductivity in iron chalcogenides.

A primary reason for the dilemma is the large run-to-run variation in properties of FeSe fabricated by PLD associated with the inherent dynamics and hyper-thermal process in the plasma plume of PLD involving multiple ion species and particulates. Previously, a crossed-beam PLD technique utilizing interacting plumes generated by two laser beams on two targets has been developed to tune the spatial profile of the particulates in the film [25–27]. In this work, we report a new method for achieving a continuous tuning of the lattice structure on a single film, achieved by a home-designed dual-beam PLD system. This technique has allowed us to synthesize FeSe films which display a continuous stretch (compression) in the out-of-plane (in-plane) lattice constant across a 3 cm width of a film on a CaF_2_ substrate. The advantage of this method is evident in the rapid delineation of a precise correlation between $T_{\mathrm{c}}$ and the lattice constant as we present below. This is in sharp contrast to a “random” distribution of the lattice constant data from a large number of individual FeSe films deposited under similar conditions with a conventional single-beam PLD process.

It has been recognized that $T_{\mathrm{c}}$ of individual FeSe films grown by PLD has a subtle dependence on the laser fluence [5]. Namely, the larger fluence seems to lead to higher $T_{\mathrm{c}}$ (Supplementary Fig. S1 in Additional file 1). In order to highlight the advantage of the plume control of the dual-beam PLD, we look at a collection of data taken from over 200 individual FeSe films made by the conventional PLD and compare them against the data from two lattice gradient films made by the dual-beam PLD, as summarized in Fig. 1. For both conventional (Fig. 1(a)) and dual-beam PLD (Fig. 1(e)), we are fabricating (00*l*)-oriented FeSe films with high crystalline quality (Supplementary Fig. S3) on CaF_2_ substrates using the same FeSe target. The key characteristic of individual FeSe films made by conventional PLD is that over up to 10 mm × 10 mm area, structural and superconducting properties are uniform, as can be gleaned from x-ray diffraction (XRD) patterns zoomed in around the FeSe (002) reflection (Fig. 1(b)) and the temperature dependence of resistance [$R(T)$] (Fig. 1(c)) measured across the sample for a typical film. In Fig. 1(d), we plot the *c*-axis lattice constant versus $T_{\mathrm{c0}}$ (zero resistance transition temperature) from 200 such individual FeSe film samples randomly selected from about 1500 experimental runs using conventional single-beam PLD performed over a three-year period. Apart from a general positive correlation between $T_{\mathrm{c}}$ and *c*-axis lattice constant (the correlation coefficient is ≈0.70 and a linear fit gives the coefficient of determination $R^{2} \approx 0.49$), no further details in the functional dependence can be extracted due to the scatter in the data. This is not surprising since the superconducting properties of FeSe films are sensitive to many factors such as strain effects, point and line defects [28–30], and the subtle day-to-day variation in fabrication conditions can influence physical properties of the resulting films in a multitude of ways.

**Figure 1:**
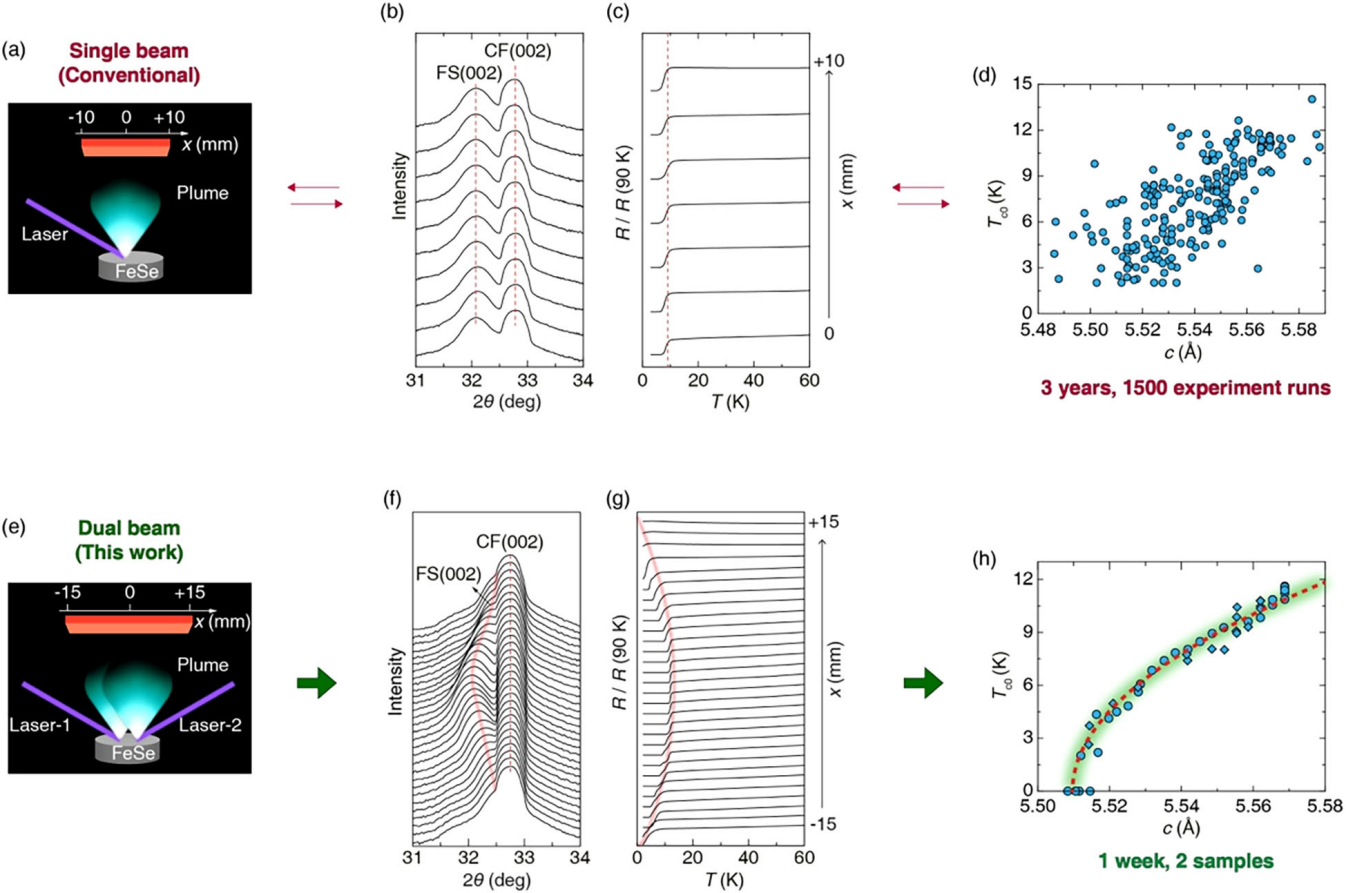
Comparison of conventional single-beam (a–d) and dual-beam (e–h) pulsed laser deposition (PLD) methods and resulting lattice constant – $T_{\text{c}}$ variation in FeSe films. (a) Schematics of the single-laser PLD method. (b) The XRD patterns for the out-of-plane (002) peak taken at equally spaced positions along the *x*-direction from a single representative film with $T_{\text{c}}$ of ${\approx} 8\text{ K}$. (c) The temperature dependence of the normalized resistance [$R(T)$] curves at different positions on the same film as in panel (b) along the *x*-direction. In panels (b) and (c), the curves are vertically shifted for clarity. The characterized region moves from the center ($x = 0\text{ mm}$) to the edge ($x = 10\text{ mm}$) of the substrate from the bottom to the top curves, as indicated by the black arrow on the right side. The XRD peak position in 2*θ* and $T_{\text{c}}$ do not change across the sample as in any typical film made by conventional PLD, as marked by the straight red dotted lines. (d) The out-of-plane lattice constant (*c*-axis) versus the superconducting transition temperature ($T_{\text{c}}$) for FeSe films made by conventional PLD. 200 sample data points plotted are selected randomly from more than 1500 samples. (e) Schematics of the dual-beam PLD method. (f) The XRD patterns for the out-of-plane (002) peak of the dual-beam FeSe film along the *x*-direction from −15 to +15 mm, encompassing the entire length of the substrate. A clear continuous shifting of the FeSe (002) peak is observed, first to lower angle approaching the middle position (0 mm), and then the shifting is reversed in a symmetrical manner. (g) The corresponding normalized $R(T)$ curves at different locations along the *x*-direction, with the highest $T_{\text{c}}$ in the middle, corresponding to the position on the film with the largest *c*-axis as well as the shortest *a*-axis. The curves are shifted vertically for ease of comparison. The red lines in panels (f) and (g), track the variations of the FeSe (002) peak and $T_{\text{c}}$, respectively. The arrow on the right side indicates the *x* coordinate positions on the film from bottom to top with 0.5 mm increment. (h) The *c*-axis lattice vs $T_{\text{c}}$ for FeSe extracted from two dual-beam PLD thin films (filled circles are extracted from panels (f) and (g)). The red dashed line is the best fit using the expression $T_{\text{c}} \propto \sqrt{c- c_{0}}$, with $c_{0} = 5.51$ Å

In contrast to a conventional PLD process involving a single laser shot on a target (Fig. 1(a)), our dual-beam deposition process employs two sliding laser beams to create a variation of beam density on a single FeSe target (Fig. 1(e)), resulting in modulation of the local properties of the deposited film across the substrate. The separation between the two laser beams is about half of the beam size (Supplementary Fig. S5). A small variation of the laser beam separation (overlap between 1/2 and 1/3 of the beam size) has no significant impact on our experimental results. As can be seen in Fig. 1(f), we are able to track the evolution of the crystal structure of the FeSe film across the 30 mm width of the substrate with an in-house x-ray diffractometer with a beam width of ${\approx} 0.4\text{ mm}$. From one side of the substrate to the other side, the FeSe (002) Bragg peak first moves to a lower angle as the substrate center position is reached, and then it shifts back away from the center (Fig. 1(f)); in contrast, the (220) peak shifts in the opposite direction [Full diffraction patterns can be found in Supplementary Fig. S3, and the evolution of the in-plane FeSe (220) peak is shown in Supplementary Fig. S4]. The evolution trend follows the profile of the dual-beam density, which is roughly symmetric about the center of the substrate (Supplementary Fig. S5). From the center to the edge, the *c*-axis parameter gradually shrinks from 5.57 to 5.51 Å, whereas the *a*-axis parameter expands from 3.73 to 3.78 Å, corresponding to the lattice constant change of ${\approx} 1.1\%$ and ${\approx} 1.3\%$, respectively.

From one such dual-beam FeSe film, $T_{\mathrm{c}}$ is mapped from a series of $R(T)$ curves obtained by patterning the sample into an array of micro-bridges. Specifically, a long conducting channel (width ${\approx} 200~\mu \text{m}$) is configured along the gradient direction of the substrate (length ${\approx} 30\text{ mm}$), with electrodes arranged alongside the channel in a Hall-bar configuration, and micro-bridges are defined by neighboring electrodes with a separation of 500 *μ*m. Along the *x*-direction defined in Fig. 1(e), $T_{\mathrm{c0}}$ first increases, reaching the maximum of approximately 12 K at the center, where the onset superconducting transition temperature is about 14 K, and then decreases toward the other end, revealing a positive (negative) correlation between $T_{\mathrm{c}}$ and the *c*-axis (*a*-axis) lattice constant. From two such films, a clear lattice-$T_{\mathrm{c}}$ relationship of FeSe has emerged (Fig. 1(h)) which can be well captured by the expression $T_{\mathrm{c}} \propto \sqrt{c- c_{0}}$, where *c* represents the *c*-axis lattice constant and $c_{0} = 5.51$ Å is a constant.

Having unmasked the fundamental relation between the *c*-axis lattice constant and $T_{c}$, we now look for its microscopic origin by performing scanning transmission electron microscopy (STEM) on a number of representative FeSe thin films with different $T_{\mathrm{c}}$’s fabricated by standard PLD according to the lattice-$T_{\mathrm{c}}$ relationship. Figures 2(a) and 2(b) show high angle annular dark field (HAADF) images of low-$T_{\mathrm{c}}$ (SC03) and high-$T_{\mathrm{c}}$ (SC09) films, respectively, along the [100] projection. Here, we use the notation SCXX to indicate a uniform film sample with $T_{\mathrm{c}} = \text{XX K}$. The HAADF image of SC09 shows a standard FeSe lattice (Fig. 2(a)), while for SC03 (Fig. 2(b)) clearly distorted regions are observed (inside region enclosed by the white dashed line). The average size of these distorted regions is approximately 5 nm. The bottom panel of Fig. 2(c) shows a magnified image of the distorted area in Fig. 2(b). On the left side, the originally overlapping Se atoms projections have split into two sets. The splitting gradually increases from left to right and eventually becomes half of the original distance between two nearest Se atoms, resulting in the observed distorted regions. The splitting of the Se atoms projections suggests the observed pattern is an overlap between two sets of lattices, i.e. the overlap between a distorted plane and a standard plane (as illustrated in Fig. 2(c)).

**Figure 2:**
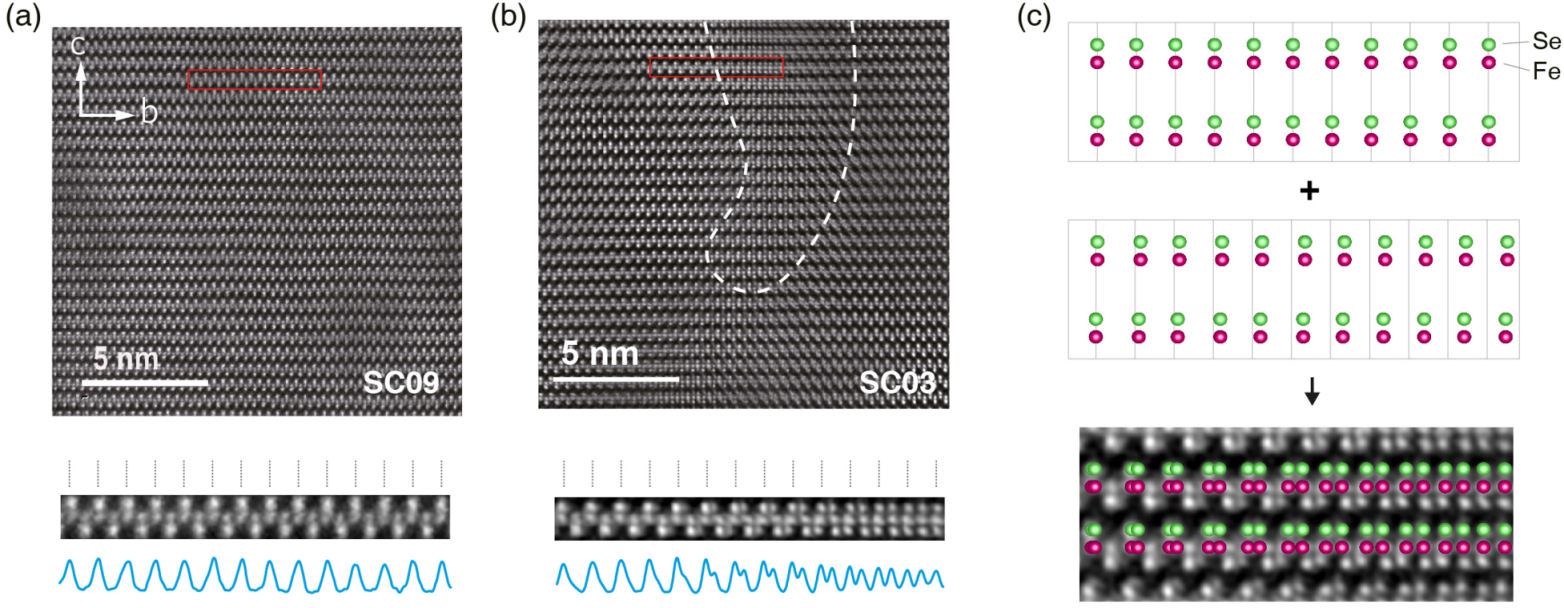
HAADF STEM images taken along the [100] zone axis for two superconducting FeSe films with different $T_{\text{c}}$’s. (a–b) Typical STEM images for samples with (a) $T_{\text{c}} = 9\text{ K}$ (SC09) and (b) $T_{\text{c}} = 3\text{ K}$ (SC03). The white dashed line in panel (b) highlights the region where the lattice is distorted, with typical dimensions of several nanometers. The bottom images show the magnifications of the red rectangles in the top images, and the blue curves indicate the contrast change across the top row of Se atoms. The grey dashed lines represent the period of the in-plane lattice. For SC09, the atoms well match the periodicity. For SC03, the left side is normal while approaching the right side the periodicity gradually becomes half, indicating lattice distortion around this region. (c) The bottom image is the magnified view of the distorted region in panel (b). The schematic picture above it reconstructs the Fe and Se atom configurations in the bottom image, and the top two pictures together illustrate that the observed lattice distortion is an overlap of a standard FeSe region (top plane) and a stretched region (second plane)

Such distortions in SC03 seemingly originate from partial edge dislocations, i.e. an extra half-unit-cell plane is inserted between two nearby (020) planes, a picture supported by the inverse fast Fourier transform (IFFT) image with (020) spots (see Supplementary Fig. S7 for details). The in-plane lattice of the distorted plane is stretched compared to that of the standard plane. Such in-plane expansion can bring about stress to the entire sample, which increases the in-plane lattice constant and decreases the out-of-plane lattice constant. We have looked at a number of STEM samples and found that the number of the distorted regions for low-$T_{\mathrm{c}}$ samples (such as SC03) is much more than that from high-$T_{\mathrm{c}}$ samples. As a rough estimate, the distorted regions occupy ${\approx} 10\%$ of the total volume of SC03 (Supplementary Fig. S8), while the high-$T_{\mathrm{c}}$ sample (SC09) is almost distortion-free.

As for the origin of these local lattice distortions, strain effects [21, 31, 32] from the substrate can be ruled out since the dual-beam FeSe film with gradient $T_{\mathrm{c}}$ is deposited on the same substrate in a single experiment, and therefore the stress is uniform across the substrate. Moreover, strain from the substrate is expected to be largely relaxed for the 200 nm thick film. One might surmise that the distribution of laser energy on the target can result in an uneven deposition rate across the substrate, which in turn can lead to density variation of lattice distortions at different locations. However, the thickness of the film is nearly constant across the 30 mm substrate, with a variation of less than 1% from the center to the edge (Supplementary Fig. S9). Thus, we also exclude the variation of deposition rate as the reason for the distortion.

Since films made by single-beam PLD do not exhibit location-dependent properties across the substrate, the reason for the distribution of the lattice parameters in the dual-beam PLD configuration must lie in the laser-material interactions. To gain insight into this phenomenon, we have applied a kinetic-fluid joint model for quantitative simulations of laser-plume dynamics created by the laser pulses. In the first step, we used the mesoscopic two-temperature molecular dynamics simulation method [33] to study the influence of the acting laser on the cluster distribution, spallation, and particle motion. This process occurs within the laser-material interaction zone, which lies close to the surface of the target, i.e. $z < 0.2~\mu\text{m}$ (*z* denotes the vertical distance from the target surface). The formation of particle clusters and voids, as well as the particle distribution are monitored and taken as the initial conditions for the plasma plume expansion in the second step, where we have adopted a plasma fluid model [34] to describe the expansion process. Details of the simulation can be found in Supplementary Note 3.

We summarize the simulation results in Fig. 3. In the plasma plume expansion stage, the appearance of plasma plume is similar to a mushroom cloud (Fig. 3(a)), formed by vortex-like convection of the heat flow colliding with the cold flow at the top. The integration of the plasma density at the top of the mushroom cloud has a uniform mass density distribution (Fig. 3(b)), namely the total amount of molecules shows little variation in the lateral direction, which explains the constant thickness observed across the substrate. The inset images of Fig. 3(c) show the mesoscopic atomic cluster distribution of the plume close to the target during the ablation process. The results are obtained by a series of molecular dynamic simulations according to the spatial profile of the laser intensity. The dimensions of these images are roughly 0.05 *μ*m in the *x*-direction and 0.2 *μ*m in the *z*-direction, and the laser intensity for obtaining each individual image is indicated by the red arrows in Fig. 3(c). In the center where the laser fluence is the highest, molecular clusters of the plume are smaller, and the size distribution is more even. In contrast, a complex structure of plume with larger clusters and voids appears away from the center with lower laser fluence. These observations are highly consistent with previous studies where particulate density is significantly reduced in the intersection area of two ablation plumes from twinned simultaneously irradiated targets [25, 35]. This non-uniform distribution of the cluster size is inherited in the plume expansion and brings about uneven formation of edge dislocations and associated lattice distortions in the deposited film, as larger clusters induce more stress during the film growth.

**Figure 3:**
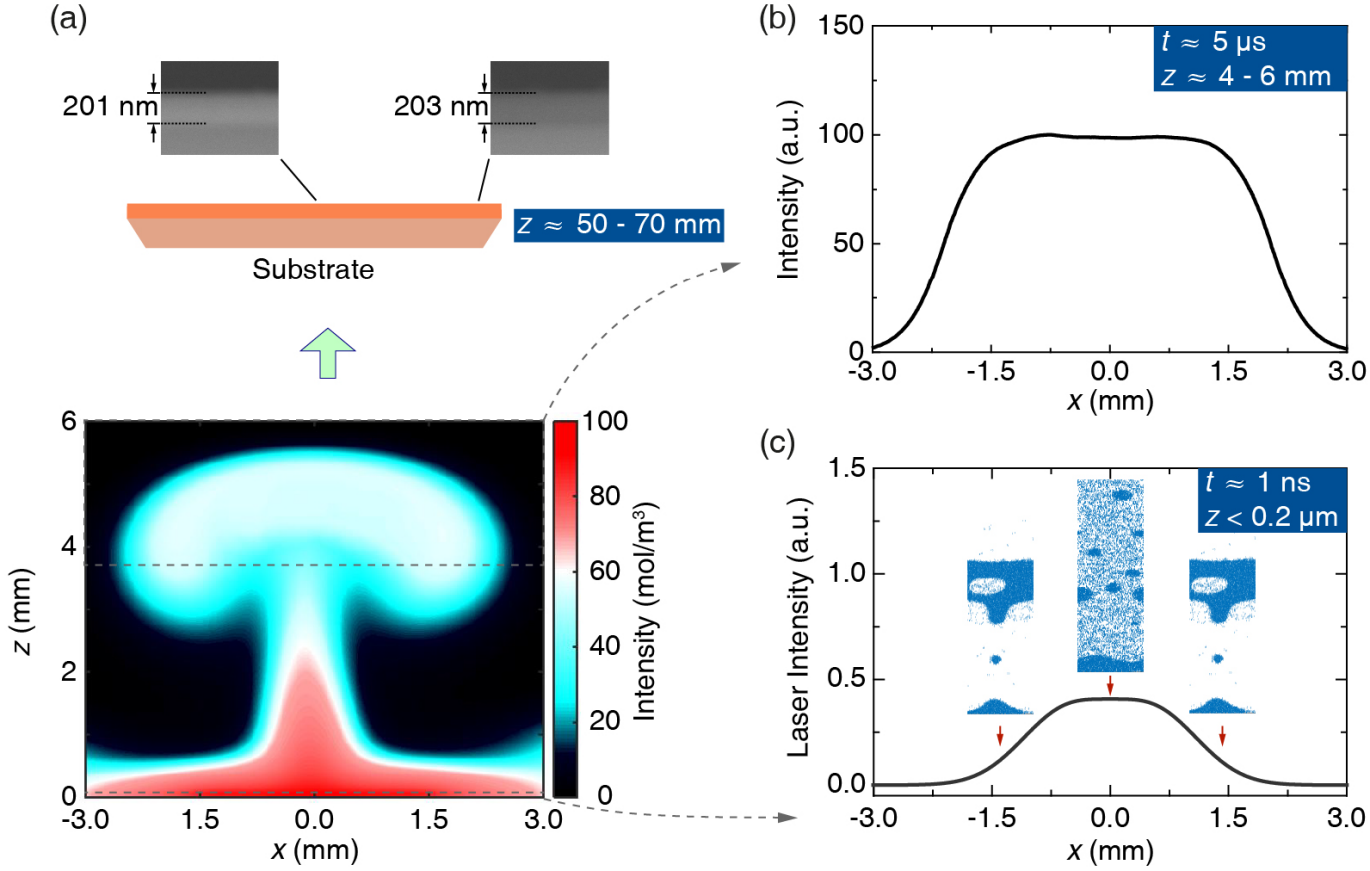
The density distribution and the morphology of atomic clusters of the plasma plume due to dual-beam laser ablation. (a) (Bottom) The density distribution of plasma above the surface of FeSe target computed by the fluid model; (Top) Cross-sectional SEM image at the center and edge of the resulted FeSe film, with their thicknesses labeled respectively. $z = 0\text{ mm}$ corresponds to the top surface of FeSe target, and the substrate are 50–70 mm above the target. (b) Integration of the plasma density at the top of the mushroom cloud in panel (a), indicated by the dashed box. The plasma density exhibits no significant change along *x*-direction, corroborating the uniform thickness of the FeSe film deposited on the substrate. (c) The intensity distribution of the dual-beam laser on the FeSe target. The inset white-blue images show distributions of mesoscopic atomic clusters evaporated from the FeSe target near the laser-material interaction zone ($z < 0.2~\mu \text{m}$ in panel (a)) obtained by the molecule dynamic simulation at three *x* locations with different laser intensities (indicated by the red arrows). The image dimensions are roughly 0.05 *μ*m in the *x*-direction and 0.2 *μ*m in the *z*-direction (limited by the number of unit cells which can be treated by the molecular dynamic simulation). Each colored pixel represents one unit cell (molecule). Particle clusters (dark blue areas) and voids (light areas) can be clearly discerned indicating that higher energy intensity leads to more uniform cluster distribution in the micrometer range near the interaction zone. If $t= 0\text{ s}$ denotes the moment the laser pulse hits the target surface, the time scale is approximately $t = 1\text{ ns}$ for panel (c) and $t = 5~\mu \text{s}$ for panel (b)

We now turn to the impact of the lattice distortion on the superconducting properties. Figure 4 shows the summary of the structural and transport properties of individual films with different $T_{\mathrm{c}}$’s, which can be mapped in a manner consistent with the lattice constant-$T_{\mathrm{c}}$ relation revealed here (inset of Fig. 4(b)). The samples exhibit high crystalline quality as manifested in the small FWHM value (≈0.6^∘^) of the rocking curves (Fig. 4(a)) and sharp superconducting transitions (Fig. 4(b) and Supplementary Fig. S11), e.g. $\Delta T_{\mathrm{c}} = 0.3\text{ K}$ for SC12. The highest $T_{\mathrm{c}}$ here is ${\approx} 14\text{ K}$, which is almost twice that of typical FeSe single crystals ($T_{\mathrm{c}} \approx 8\text{ K}$) [7].

**Figure 4:**
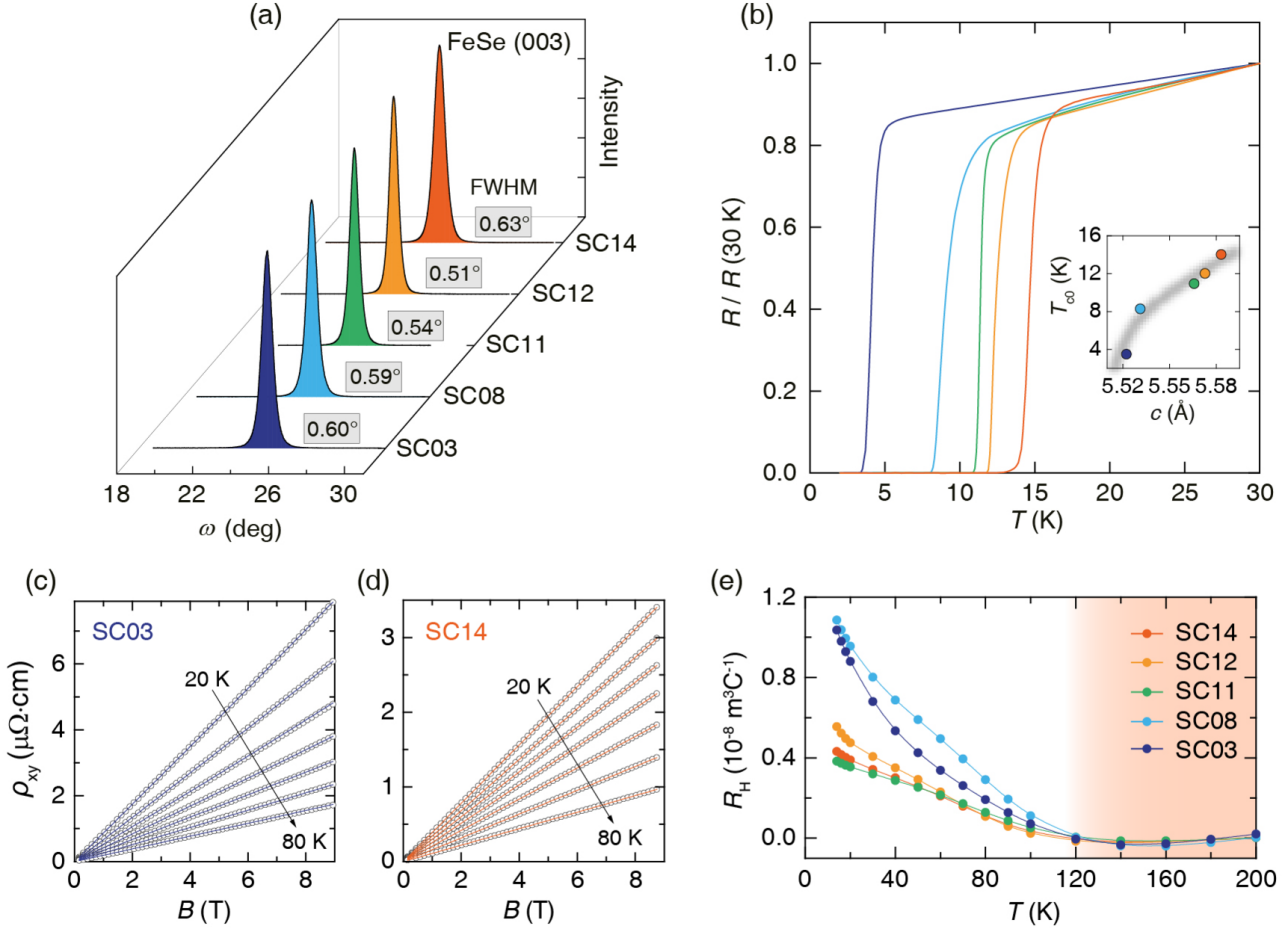
Transport properties of five representative uniform FeSe thin films with different $T_{\text{c}}$’s (samples denoted as SC03, SC08, SC11, SC12, and SC14 have $T_{\text{c}} = 3, 8, 11, 12, \text{and }14\text{ K}$, respectively). (a) X-ray rocking curves of the FeSe (003) peak. (b) The temperature dependence of the normalized in-plane resistivity. Inset: The mapping of the *c*-axis lattice and $T_{\text{c}}$ relation of the uniform FeSe thin films (colored dots) to the lattice constant – $T_{\text{c}}$ relation (grey line) we uncovered here (i.e. $T_{\text{c}} \propto \sqrt{c- c_{0}}$). (c–d) The Hall resistivity $\rho _{\text{xy}}(B)$ at 10 K intervals for two samples: SC03 (c) and SC14 (d). Here, the symbols are experimental data and the solid lines are the linear fits. (e) The corresponding temperature dependence of the Hall coefficients [$R_{\text{H}}(T)$] for different samples. All the $R_{\text{H}}$ (*T*) curves overlap above $T^{*} \sim 120 \pm 10\text{ K}$, indicated by the orange shadow area, but diverge considerably below $T^{*}$

The Hall resistivity $\rho _{\mathrm{xy}}$ is proportional to the magnetic field (*B*) from 200 to 20 K (Figs. 4(c) and 4(d) & Supplementary Fig. S12), namely the Hall coefficient $R_{\mathrm{H}}(B)$ ($=\rho _{\mathrm{xy}}/B$) is field independent. $R_{\mathrm{H}}(T)$ shows a sign change from negative to positive at a crossover temperature $T^{*} \approx 120 \pm 10\text{ K}$ for all samples (Fig. 4(e)). Remarkably, the $R_{\mathrm{H}}(T)$ curves of all samples overlap at $T > T^{*}$. Our first-principles calculations indicate that 0.5% deviation in stoichiometry in FeSe would lead to doping effects, leading to significant change in the Hall coefficient. This is contrary to the present observation of $R_{\mathrm{H}}(T)$ curves collapsing to the same temperature dependence. Thus, we conclude that there is no significant off-stoichiometric effect in our study (see Supplementary Note 5 for more details). In addition, neither energy dispersive x-ray spectroscopy nor the inductively coupled plasma techniques showed discernible stoichiometric change among the films, within the error bar of 2%. Note that the characteristic temperature $T^{*}$, slightly higher than the structural transition temperature ($T_{\mathrm{s}} \approx 90\text{ K}$), is consistent with the previously reported temperature for the band splitting associated with the nematic phase [36, 37], which may explain the divergence of $R_{\mathrm{H}}(T)$ curves below $T^{*}$.

Based on our experimental observations, we consider an explanation for the continuous tuning of $T_{\mathrm{c}}$ resulting from the electronic structure modulation due to lattice constant change. In FeSe superconductors, the hole Fermi pockets around **Γ** are known to be dominated by the $d_{\mathrm{xz}}/d_{\mathrm{yz}}$ orbital bands (the $d_{\mathrm{xy}}$ band is below the Fermi level) [38–40], while the electron Fermi pockets around **M** are attributed to all three $t_{\mathrm{2g}}$ orbitals [36, 41]. With a decrease (increase) in $a(c)$-axis, the Se height increases. Phenomenologically, since the diagonal hopping between $d_{\mathrm{xy}}$ orbitals is sensitive to the Se height (i.e. the vertical distance from the Se atoms to the Fe plane) while $d_{\mathrm{xz}}/d_{\mathrm{yz}}$ is not, a small variation in the Se height will mainly affect the $d_{\mathrm{xy}}$ bands. To substantiate this hypothesis, we have performed first-principles band structure calculations of FeSe in the tetragonal phase, directly incorporating three sets of lattice parameters from three positions on our dual-beam PLD film (details of the calculation can be found in Supplementary Fig. S13 and Note 5). With decreasing (increasing) *a*-axis (*c*-axis) lattice parameter, the most noticeable change in the electronic structures takes place in the $d_{\mathrm{xy}}$ band: the $d_{\mathrm{xy}}$ band shifts up in energy around **Γ** and shifts down around **M**, while $d_{\mathrm{xz}}/d_{\mathrm{yz}}$ bands exhibit little change. It has been reported that the increased weight of the $d_{\mathrm{xy}}$ pocket at the Fermi level from the downshift of the $d_{\mathrm{xy}}$ band around **M**, together with the reduced *d*–*p* coupling derived from the lattice modulation which affects the spin fluctuations [42], will weaken the nematicity [43]. A number of experimental observations have previously pointed to the presence of competition between nematicity and superconductivity [16, 44, 45]. Thus, the suppression of nematicity can naturally lead to the enhancement of superconductivity.

Another consequence of the decrease in the in-plane lattice constant in FeSe is phonon hardening and the enhanced the electron-phonon interaction. It has been argued that phonons are likely playing a critical role in FeSe thin films, especially in the $T_{\mathrm{c}}$ enhancement of single layer FeSe films on SrTiO_3_, where the electron-phonon coupling originating from either FeSe itself or across the interface has attracted significant attention [41, 46, 47]. In this regard, the enhancement of the electron-phonon interaction could lead to the boosting of superconductivity. It is also interesting to note that the isotope effect [48] in conventional superconductors has the form $T_{\mathrm{c}} \propto (1/M)^{1/2}$ (*M*: atomic mass) [49], which bears a resemblance to the quantitative relation between *c*-axis and $T_{\mathrm{c}}$ observed here, namely, $T_{\mathrm{c}} \propto ( c- c_{0} )^{1/2}$. Since the lattice vibrations are modulated by the atomic mass and lattice parameters, whether there exists a more fundamental link between the two relationships is perhaps an intriguing question for further investigation.

In summary, a unique dual-beam PLD approach has given us a panoramic view of the $T_{c}$ – lattice parameter relationship in FeSe thin films. The atomic-level microstructural investigation coupled with a theoretical analysis paints the picture of local lattice distortion resulting in electronic structure modulation, which in turn dictates the delicate balance between nematicity and superconductivity. The quantitative relation observed here can place explicit constraints on theoretical models describing the mechanism of superconductivity in FeSe.

### Supplementary Information

Below is the link to the electronic supplementary material.

(PDF 2.1 MB)

## Data Availability

The data that support the findings of this study are available in the paper. Additional data are available from the corresponding authors upon reasonable request.
