# Supplementary material for: The origin of the large Tc variation in FeSe thin films probed by dual-beam pulsed laser deposition
Source: Quantum Front. 2024 Jun 8;3(1):12. doi: 10.1007/s44214-024-00058-0 (PMC11161545; doi:10.1007/s44214-024-00058-0)
Supplement: Supplementary file 1 — (PDF 2.1 MB) [file 44214_2024_58_MOESM1_ESM.pdf]

Supplementary Information

The origin of the large T_c variation in FeSe thin films probed by dual-beam pulsed laser deposition

Zhongpei Feng[1,2,3*], Hua Zhang[4*], Jie Yuan[1,3,5], Xingyu Jiang[1,3], Xianxin Wu[6], Zhanyi Zhao[1,3], Qiuhao Xu[1,3], Valentin Stanev[7,8], Qinghua Zhang[1,3], Huaixin Yang[1,3], Lin Gu[1,3], Sheng Meng[1,3], Suming Weng[9,10], Qihong Chen[1,3†], Ichiro Takeuchi[7,8†], Kui Jin[1,2,3,5†], and Zhongxian Zhao[1,2,5]

[1]Beijing National Laboratory for Condensed Matter Physics, Institute of Physics, Chinese Academy of Sciences, Beijing 100190, China.

[2]Songshan Lake Materials Laboratory, Dongguan, Guangdong 523808, China.

[3]University of Chinese Academy of Sciences, Beijing 100049, China.

[4]Center for Intense Laser Application Technology, Shenzhen Technology University, Shenzhen 518118, China

[5]Key Laboratory for Vacuum Physics, University of Chinese Academy of Sciences, Beijing 100049, China.

[6]CAS Key Laboratory of Theoretical Physics, Institute of Theoretical Physics, Chinese Academy of Sciences, Beijing 100190, China

[7]Department of Materials Science and Engineering, University of Maryland, College Park, MD 20742, USA.

[8]Maryland Quantum Materials Center, University of Maryland, College Park, MD 20742, USA.

[9]Key Laboratory for Laser Plasmas (MoE), School of Physics and Astronomy, Shanghai Jiao Tong University, Shanghai 200240, China

[10]Collaborative Innovation Center of IFSA, Shanghai Jiao Tong University, Shanghai 200240, China

Supplementary Figures

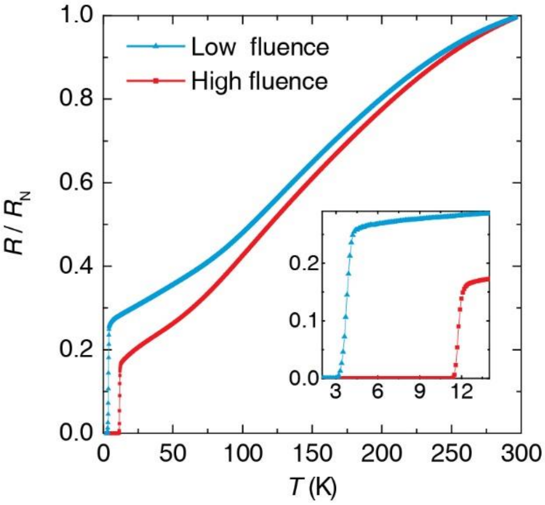

Figure S1 | Temperature dependence of the normalized resistance for conventional single-beam FeSe films deposited with different laser fluences. R_N is defined as the resistance at 300 K. The high laser energy density (3 J/cm^2) is two times that of the low one. T_{c0} of the as-prepared FeSe films is enhanced by a factor of ≈ 4 as can be seen in the inset.

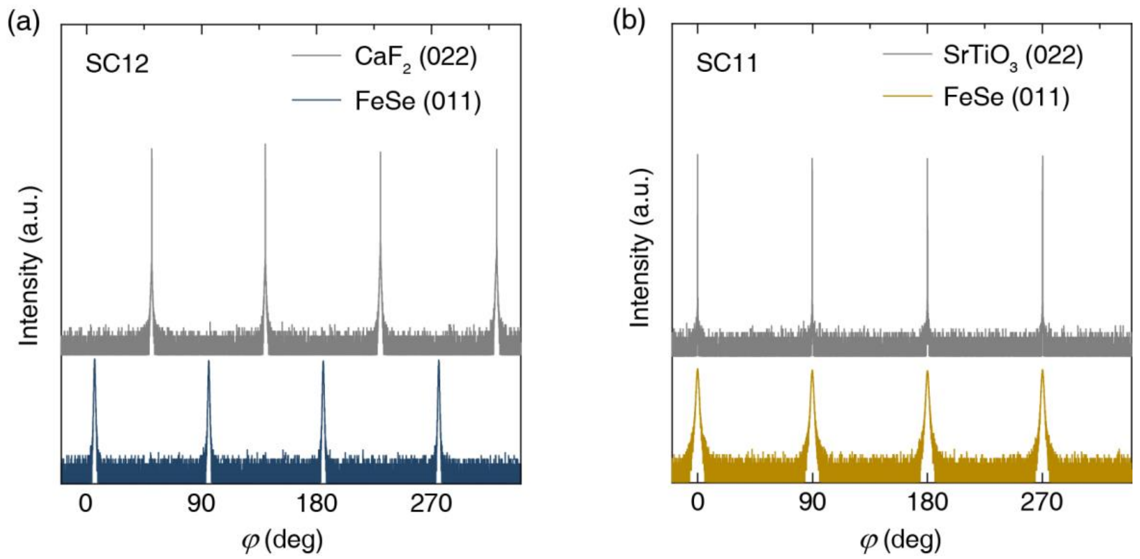

Figure S2 | X-ray φ scan of FeSe films deposited with conventional single-beam PLD. (a) and (b), The x-ray φ scan diffraction patterns for SC12 [deposited on CaH_2 (001)] and SC11 [deposited on $SrTiO_3$ (100)], respectively. All FeSe films here exhibit a clear four-fold symmetry.

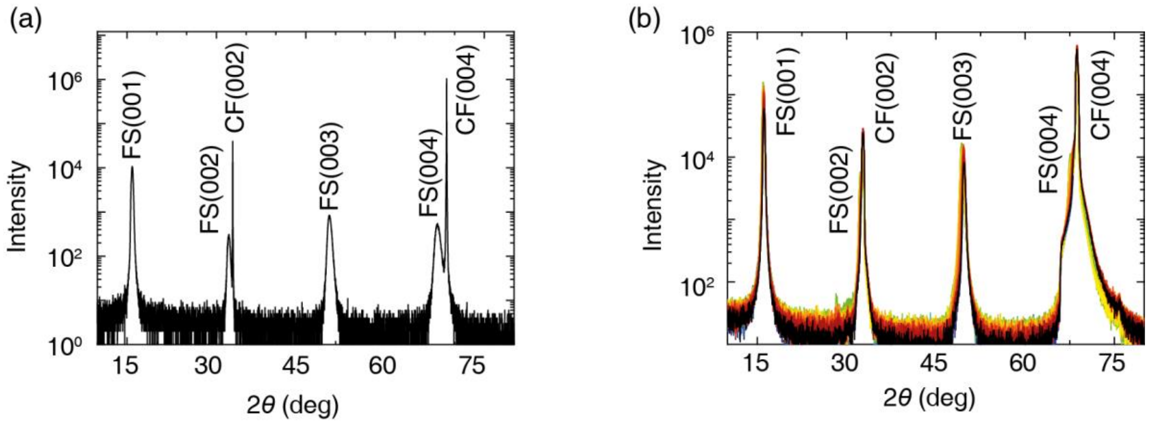

Figure S3 | θ-2θ diffraction patterns. (a) FeSe film made by the conventional single-laser PLD method. (b) FeSe film made by the dual-beam PLD method. “FS” and “CF” stand for FeSe and CaF_2 (substrate), respectively. Different colors in panel (b) represent data taken at different locations of the film using an x-ray beam with a width of ≈ 0.4 mm. Detailed evolution of the peak as a function of the position is shown in Fig. 1(f) of the main text.

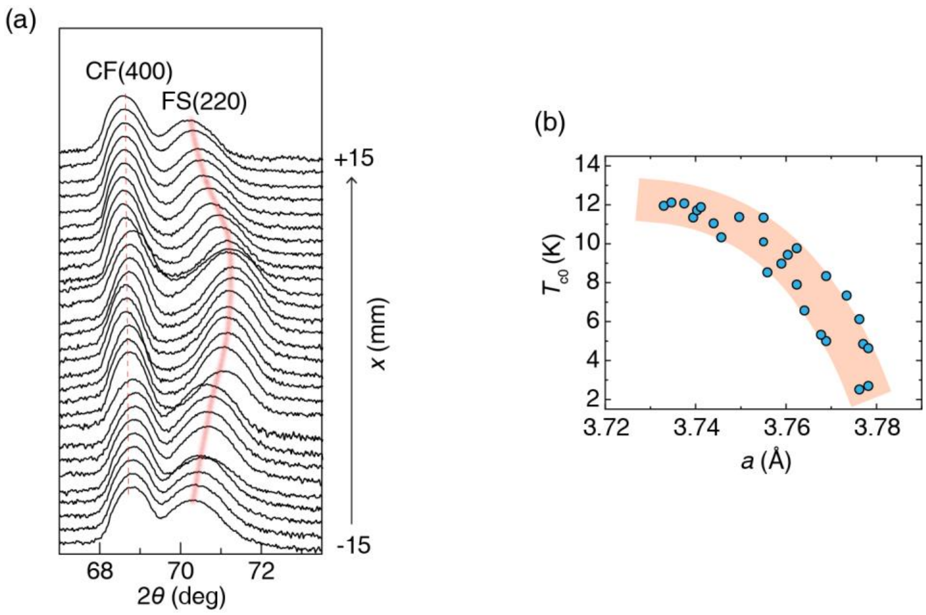

Figure S4 | Evolution of the in-plane lattice parameter of the dual-beam PLD FeSe film across the substrate. (a) The XRD patterns of the (220) peak along the *x*-direction (as denoted in Fig. 1(e) of the main text) from -15 to +15 mm. The grazing-incident x-ray beam is aligned parallel to the *ab*-plane of the FeSe film. A clear continuous shift is observed, first to higher angle approaching the middle and then the shifting is reversed in a symmetrical manner. (b) The correlation between the *a*-axis lattice constant (extracted from panel (a)) and T_c.

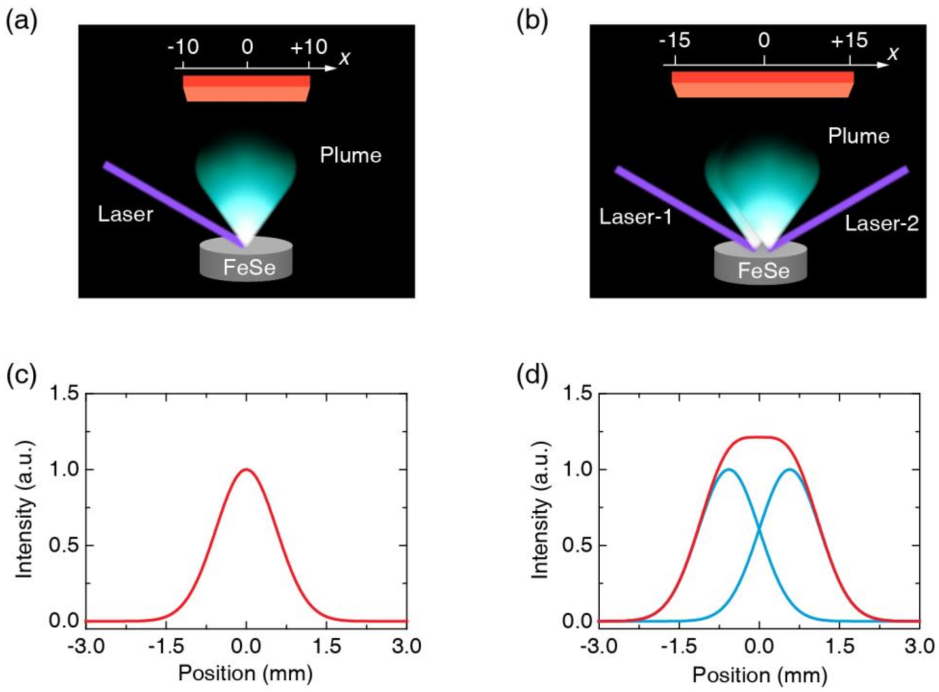

Figure S5 | Comparison of the laser energy distribution for the single- and dual-beam PLD. (a-b) Single- (a) and dual- (b) beam PLD configurations. (c-d) The laser energy distribution for the single- (c) and dual- (d) beam configurations.

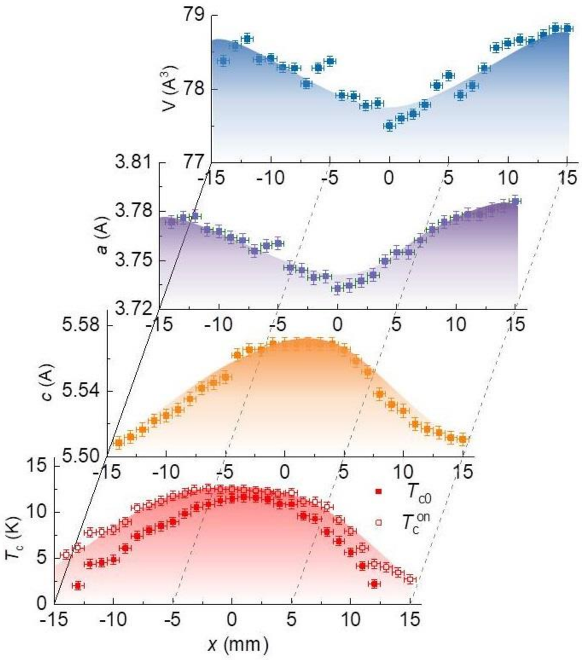

Figure S6 | Variation of T_c and lattice parameters across the dual-beam FeSe film. From bottom to the top, panels show the position (*x*-direction) dependence (across the substrate) of T_c, *c*-axis, *a*-axis and unit cell volume (*V*).

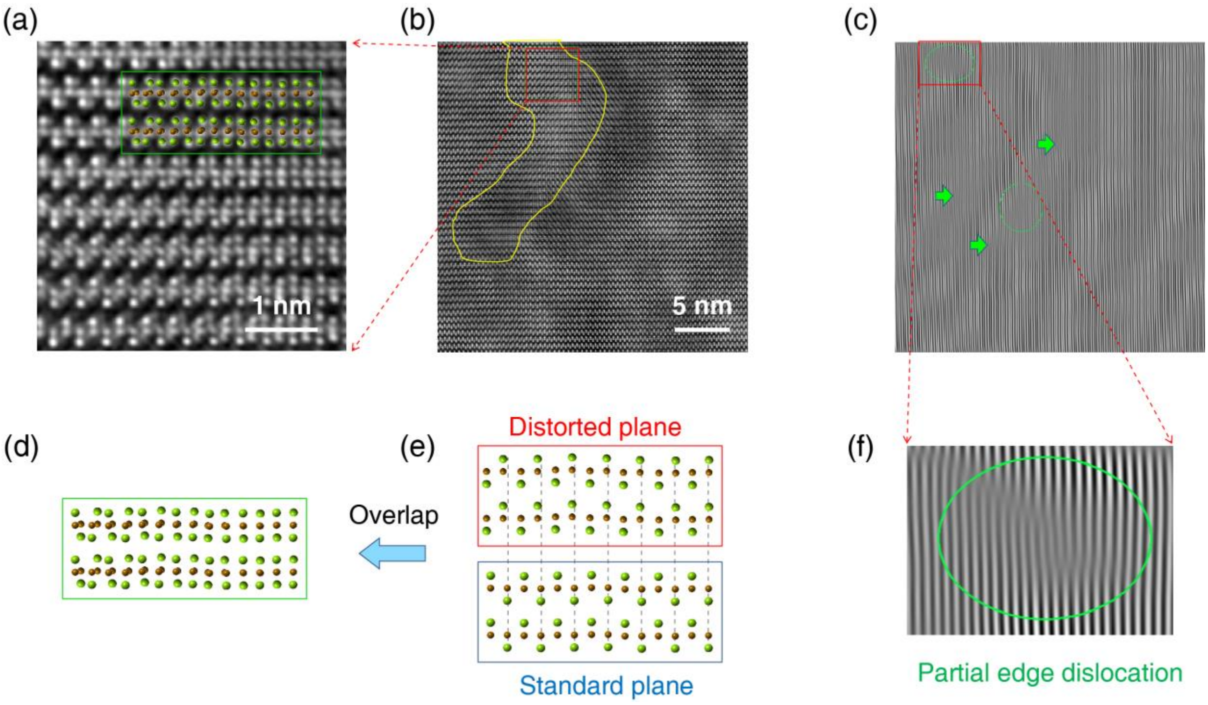

Figure S7 | Schematic illustration of the distorted region and its relation with the variation of the lattice constants. Panel (a) is a magnified image of the area enclosed by the red box in panel (b), which is the HAADF image of the sample with T_c of 3 K along the [100] projection. Panel (c) is the inverse fast Fourier transform (IFFT) image with (020) spots, and panel (f) is the blow-up image of the area enclosed by the red box in panel (c). Panels (c) and (f) show there exist partial edge dislocations (i.e. an extra half-unit-cell plane inserted between two nearby planes, highlighted by the green circle and arrows in panel (c)), which result in distorted (020) planes. While these distortions are a few nanometers in size, the typical thickness of our STEM specimen is 30 – 50 nm, therefore the projection we observe always include contributions from both distorted and undistorted planes. Panels (d) and (e) schematically illustrate that the distorted region is the overlap between a distorted plane and an undistorted plane. The in-plane lattice of the distorted plane is stretched compared to that of the standard plane. Such in-plane expansion introduces stress in the entire sample, leading to the increase of in-plane lattice constant and decrease of out-of-plane lattice constant.

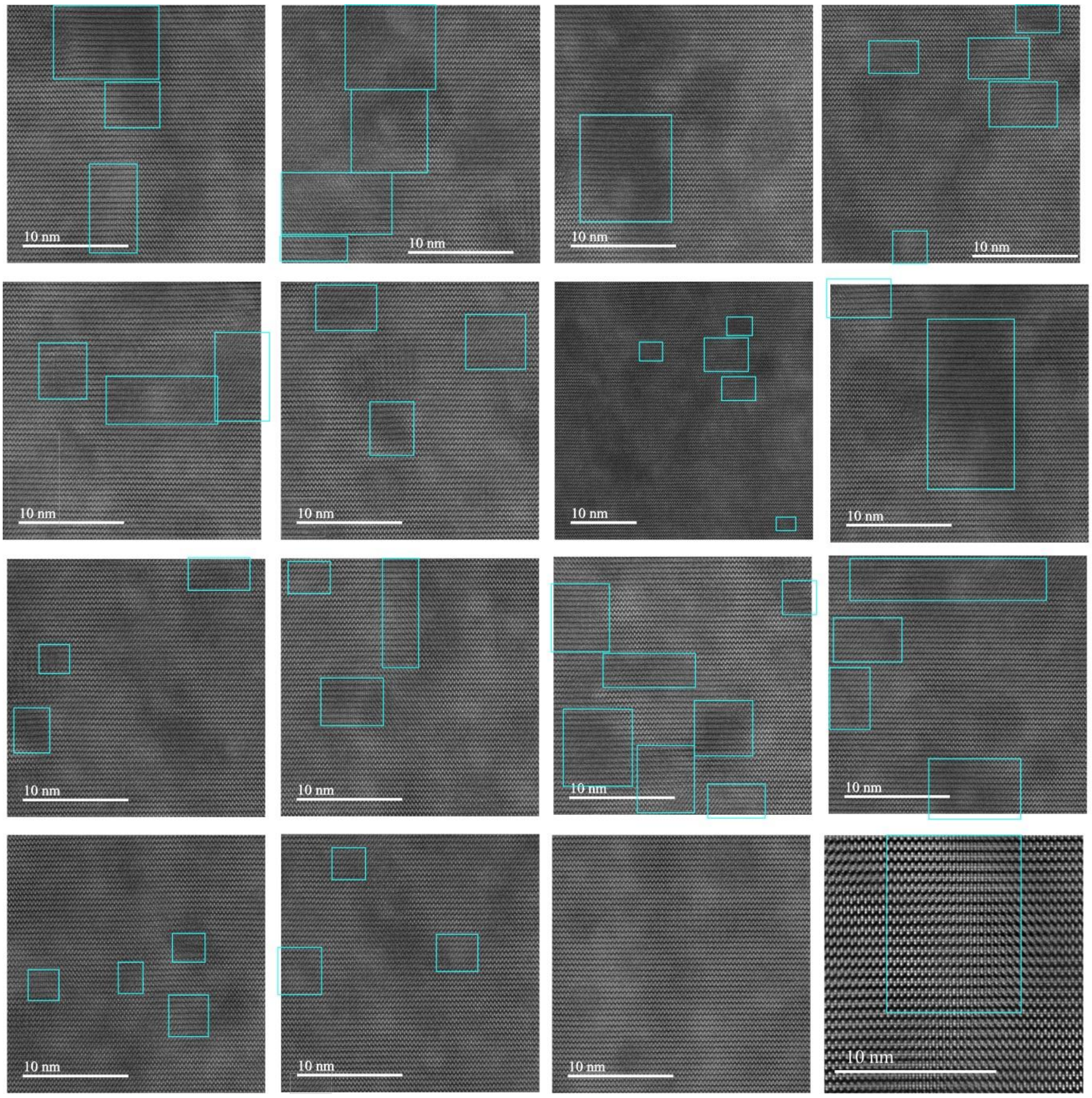

Figure S8 | Statistics of the distorted areas observed in a low-T_c FeSe film deposited by conventional single-beam PLD. The boxes roughly capture the distorted regions in each image. Overall, the distorted regions occupy around 10% of the total volume.

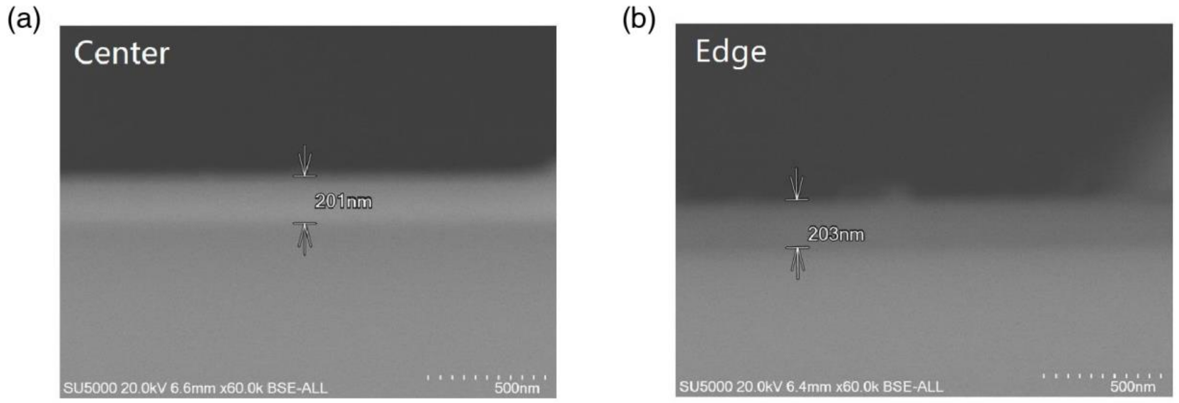

Figure S9 | Thickness determination for a dual-beam FeSe film. (a-b) The scanning electron microscope (SEM) images for the dual-beam FeSe film at the center (a) and edge (b) of the substrate, separated by ≈ 15 mm. The thicknesses (labeled in the figures) are 201 (± 5) and 203 (± 5) nm for panels (a) and (b), respectively, showing little variation from the center to the edge. This result confirms that the thickness of the dual-beam FeSe film is uniform across the substrate.

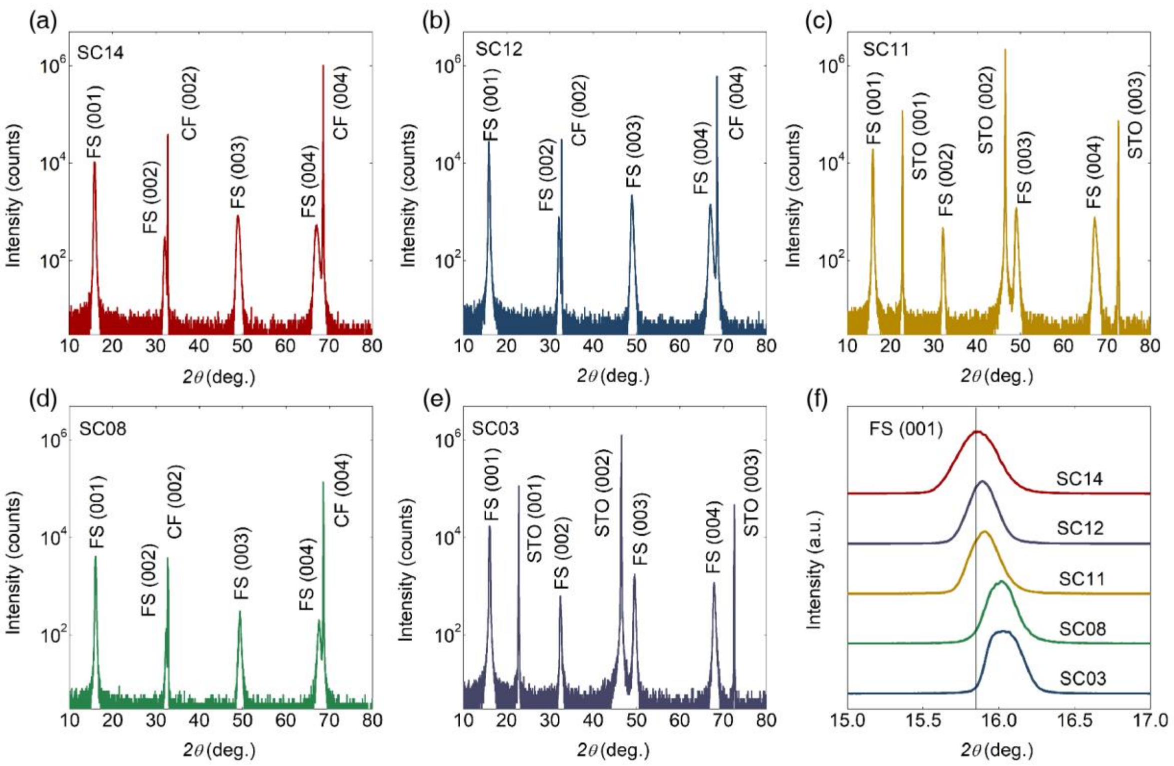

Figure S10 | X-ray analysis of individual FeSe films made via standard single-beam PLD. (a-e) θ-2θ diffraction patterns of FeSe films (samples SC14, SC12, SC11, SC08, and SC03). Here, FS (00*l*), CF (00*l*), and STO (00*l*) represent Bragg diffraction peaks of β-FeSe, CaF_2 and $SrTiO_3$ substrates, respectively. In all diffraction patterns, only (00*l*) peaks are observed for FeSe, indicating high crystallinity along the *c*-axis. (f) Zoom-in of the FeSe (001) peak.

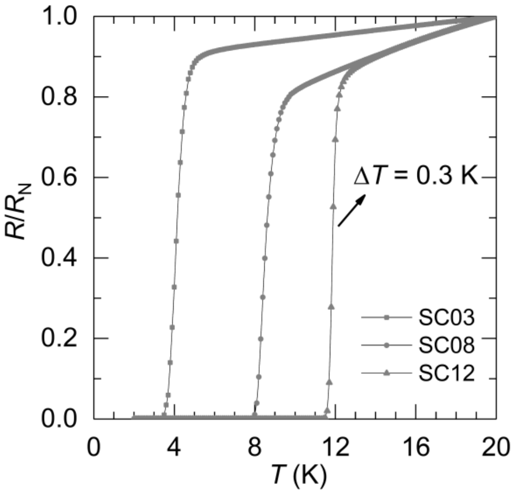

Figure S11 | Temperature dependence of the normalized resistance of FeSe films made by single-beam PLD. R_N corresponds to the resistance at 20 K. The samples show very sharp superconducting transitions (e.g. $\Delta T \approx 0.3$ K for the SC12 sample), suggesting the high qualities of the FeSe films.

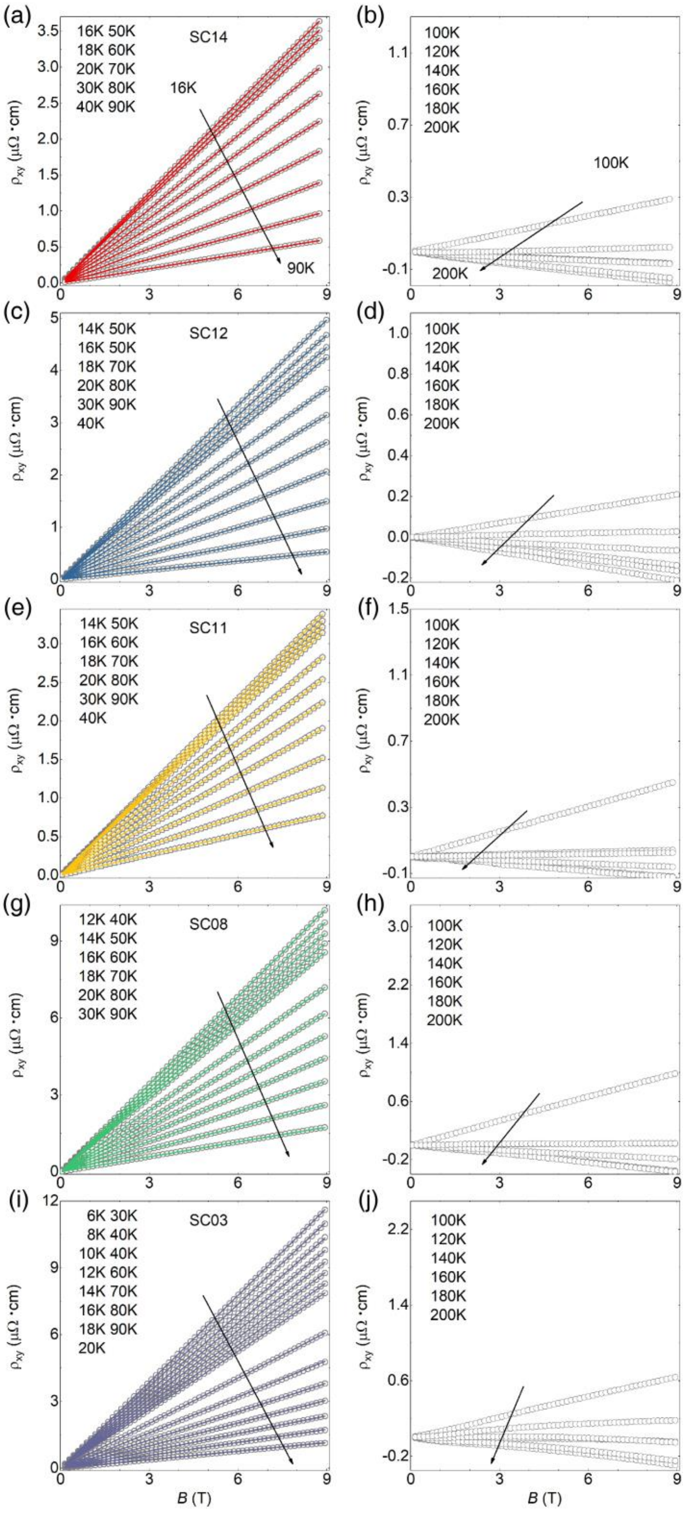

Figure S12 | The normal-state Hall resistivity $\rho_{xy}(B)$ for five FeSe films (SC14, SC12, SC11, SC08 and SC03) made by the single-beam PLD. The Hall resistivity is proportional to the magnetic field up to 9 T at all measured temperatures from 200 to 20 K, indicating that the Hall coefficient is field independent.

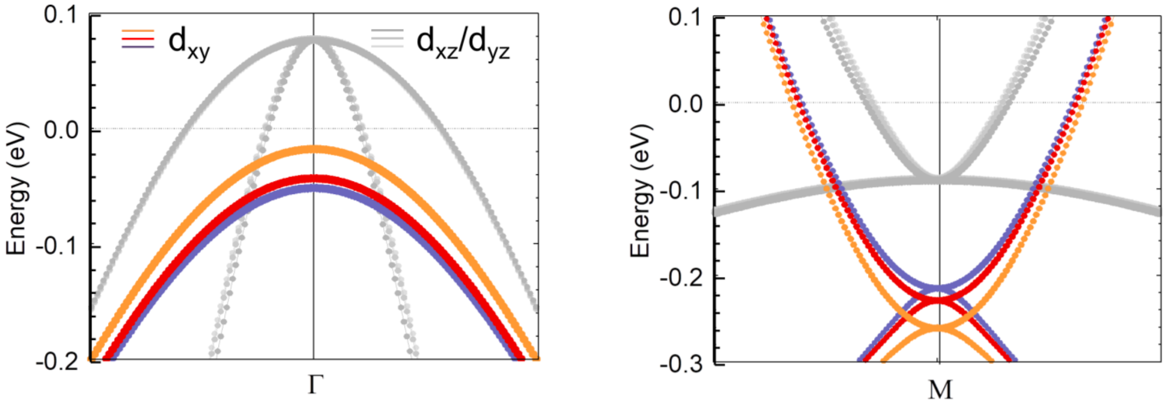

Figure. S13 | Electronic band structure of FeSe with varying lattice parameters. Band structures around **Γ** and **M** points calculated from our experimental crystal lattice parameter data taken at room temperature. Here, we show band structures based on three sets of lattice constants (Supplementary Note 5): $a_{\mathrm{purple}} > a_{\mathrm{red}} > a_{\mathrm{orange}}$ and $c_{\mathrm{purple}} < c_{\mathrm{red}} < c_{\mathrm{orange}}$. The d_{xy} orbitals are below the E_F around **Γ**, but pass through the E_F around **M**. With the lattice modulation, the most noticeable change in the electronic structures takes place in the d_{xy} band: the d_{xy} band shifts up in energy around **Γ** and shifts down around **M**, while d_{xz}/d_{yz} bands exhibit little change.

Supplementary Note 1. Samples fabrications

Individual thin films deposited by standard single-beam PLD. The β-FeSe thin films were grown on (00l)-oriented CaF_2 or $SrTiO_3$ substrates by standard pulsed laser deposition (PLD) using a KrF excimer laser (λ = 248 nm) at the deposition substrate temperature of 350 ℃. The thicknesses of all films are ≈ 160 nm. More details about the fabrication process of the thin films can be found in a previous work [*Sci. Rep.* **8**, 4039 (2018)].

Dual-beam PLD thin films. In contrast to the standard single-beam PLD technique, the laser beam was separated into two beams via reflecting mirrors in the light path with a low separation angle. As the laser pulse strikes the target, a modified plume is excited (See Supplementary Fig. S5 for the laser energy distribution). The CaF_2 substrate, with a size of 30×5 mm^2, is mounted on the heater. The target-substrate distance ranges from 50 to 70 mm for optimal films.

Supplementary Note 2. Film characterizations

Structure and surface analyses. X-ray diffraction (XRD) was performed in an x-ray diffractometer with two Ge (220) single crystals. For the FeSe films deposited with dual-beam PLD, the 0.4 mm wide x-ray beam was set to scan the film along the ***x*** direction from 0 to 30 mm (+/- 15 mm in Fig. 1). At each position, the θ-2θ pattern is taken from 10° to 80°.

Transport measurements. The transport properties of FeSe films were characterized in standard Hall bar patterns made by a focused ion beam (FIB). The dimensions of the Hall bar are 100 μm in length, 50 μm in width and 160 nm in thickness. Transport measurements were performed in a Physical Property Measurement System (PPMS) with the standard four-probe method.

Microstructure measurements. Selected area electron diffraction (SAED) patterns and high angle annular dark field (HAADF) scanning transmission electron microscope (STEM) images were obtained on a JEOL ARM200F TEM equipped with probe-forming spherical-aberration corrector. Cross-sectional TEM specimens were prepared using a FIB system.

Supplementary Note 3. Details of the laser-plume simulation

In the first step, the laser ablation of the bulk target is simulated with the hybrid model consisting of the molecular dynamics (MD) and a two-temperature model (TTM) (1). In this model, the time evolution of the lattice and electron temperatures is described by two coupled

continuum equations that account for the laser energy deposition, electron heat conduction, and electron-lattice energy exchange. The cells for the discretization of the TTM are interpolated to the associated MD system, and the local averaged temperature is used in the equations motion of the atoms. The set of equations used for the first step are

$$\text{TTM:} \quad C_e(T_e)\frac{\partial T_e}{\partial t} = \nabla \cdot [K_e(T_e, T_l)\nabla T_e] - g(T_e - T_l) + S_{laser}(\vec{r}, t), \tag{1}$$

$$C_l(T_l)\frac{\partial T_l}{\partial t} = \nabla \cdot [K_l(T_l)\nabla T_l] + g(T_e - T_l), \tag{2}$$

$$\text{MD:} \quad m_i d^2\vec{r}_i/dt^2 = \vec{F}_i + \xi m_i \vec{v}_i^{jh}, T_l = \sum m_i (v_i^{th})^2/(3k_B N_{cell}), \tag{3}$$

Here, S_{laser} represents the laser source, C_e is the electron specific heat, C_e is the lattice specific heat, g is lattice-electron coupling factor, $K_e(K_l)$ is the electron (lattice) thermal conductivity described by the Drude model. In the MD system, N_c is the number of cells and the Newton's equations of motion of atoms have the thermal velocity friction term with ξ the friction constant for the energy dissipation. In the simulation, the processes of melting as well as the ablation are confined within the MD region of the model.

In the second step, we assume that the plume will evolve from a non-equilibrium state to an equilibrium state across the thin Knudsen layer. The plume expansion is described by the fluid dynamics with the governing equations:

$$\frac{\partial \rho_g}{\partial t} + \nabla \cdot (\rho_g \vec{U}) = 0, \tag{4}$$

$$\frac{\partial \rho}{\partial t} + \nabla \cdot (\rho \vec{U}) = 0, \tag{5}$$

$$\rho(\frac{\partial \vec{V}}{\partial t} + \nabla \cdot (\vec{U}\vec{U})) + \nabla p_{\text{evap}} = 0, \tag{6}$$

$$\rho C_p(\frac{\partial T}{\partial t} + \nabla \cdot (\vec{U}T)) - \nabla \cdot (k\nabla T) = 0, \tag{7}$$

where ρ_g denotes the density of ambient gas, ρ is the plume density, $\vec{U}$ is the velocity of the flume, p_{evap} is the total pressure of the ambient gas and the plume vapor. In order to obtain the boundary condition, the spatial and velocity distributions of atoms calculated by the MD-TTM simulation within several hundred-nanometer scales are mapped onto the Knudsen layer of the fluid model. In this mapping, the thermal evaporation model (2) is used , in which the vapor pressure at the surface is obtained from the Clasius-Clapeyron equation. We also follow the conservation of mass, momentum, and energy across the Knudsen layer when applying the boundary conditions for the fluid model.

In addition, we have performed first-principles dynamics simulations of bulk FeSe under intense laser irradiation based on real-time time-dependent density functional theory.

We choose a (2×2×2) supercell with 32 Fe and Se atoms. The maximum field strength of the laser pulse ranges from 0.03 to 0.3 V/Å with a pulse width of 50 fs and the laser wavelength of 800 nm in our microscopic dynamics simulations of laser-matter interactions. We find that at the critical laser fluence when there are about 0.35 electrons per atom being photoexcited, the energy deposition from the laser pulse to the material is about 1.05 eV per atom, which induces an ultrafast nonthermal melting of bulk FeSe at a relatively low lattice temperature of about 800 K. This results in FeSe in a quasi-liquid state with fast diffusing ions (thus resembling a plasma state). Furthermore, we find that when the FeSe material is ionized by about 0.1 electron per atom, the FeSe lattice is largely distorted and the diffusion coefficient of Fe and Se ions is dramatically increased compared to the bulk material at the similar temperature under equilibrium.

Supplementary Note 4. Extension of the dual-beam PLD technique

Lattice dislocations are commonly observed in functional materials, which develop with time and are intimately related to aging of materials. Our synthesis technique provides a convenient platform for studying the aging effect as a function of space rather than time. The Coulomb interactions, which can induce metal-insulator transition or superconductor-insulator transition, are very sensitive to variation of the lattice field. Previous studies on these effects were largely based on examining naturally formed lattice distortions, whereas the dual-beam PLD technique introduced here provides a convenient platform for manipulating lattice distortions and studying the related properties. In addition, this technique is easy to implement and generalize to other materials systems. As a demonstration, we have also incorporated it into fabrication of $BaTiO_3$, a ferroelectric material. We opt not to go into the details of this $BaTiO_3$ study here since it is entirely outside the scope of the current work. We would just point out that a similar lattice variation behavior has been observed there: the lattice constant smoothly varies from the center to the edge of the substrate, showing a ≈ 1.5% overall change. While this lattice-constant gradient $BaTiO_3$ film is still under investigation, it unambiguously establishes the wide applicability of the present dual-beam approach.

Supplementary Note 5. DFT calculations

In order to rule out the possibility that the film stoichiometry is continuously changing across the dual-beam PLD film, we have performed first-principles calculations to look at the interplay between minute change in structure and stoichiometry. Based on the theory of quantum stress, a small amount of excess Fe atoms in FeSe triggers n-doping in the compound, and such a charge rebalancing could modify the lattice parameter of the system (3,

4). The Vienna ab initio software package (5) is employed to perform the DFT calculations (6). The projector augmented-wave method is used to describe the wave functions near the core. The generalized gradient approximation (7) within the Perdew–Burke–Ernzerhof (PBE) (8) parameterization is employed as the electron exchange-correlation function. The primitive cell of FeSe (space group *P4/nmm*) is used for calculations with the Brillouin zone sampling of 6×6×4. DFT-D2 treatment is employed to correct for the van der Waals interactions in layered FeSe (9). All magnetic ions are initialized ferromagnetically, and the cell shape, volume, and atomic positions are fully optimized throughout this work. After the structural optimization, the final structure of the slightly electron-doped FeSe shows that there is no spin polarization when the doping level is low. A small amount of excess Fe in the composition will lead to electron doping in the compounds. Thus, a 0.5 % increase in Fe concentration in composition can be associated with a 0.05 Å lattice expansion in *c* direction. As mentioned in the main text, such an electron-filling picture can neither explain how the value of the Hall coefficients for different samples here overlap with each other above T^* (as seen in Fig 4(e)), nor be the reason for the hole carrier concentration remaining almost constant. Thus, we rule out the composition change as the cause of the observed lattice constant change and the concomitant T_c variation across the FeSe film deposited by the dual-beam PLD.

For band structure calculations, the cutoff energy of 500 eV is used for expanding the wave functions into plane-wave basis. In the calculation, the Brillouin zone is sampled in the $\boldsymbol{k}$ space within Monkhorst-Pack scheme (10). The number of the $\boldsymbol{k}$ points is 11×11×7. We relax internal atomic positions, and forces are minimized to less than 0.01 eV/Å in the relaxation. The adopted lattice constants are: a = 3.773 Å, c = 5.505 Å for purple lines, a = 3.763 Å, c = 5.530 Å for red lines and a =3.733 Å, c = 5.562 Å for orange lines in Fig. S13.

The key findings are as follows: with decreasing *a*-axis parameter and increasing *c*-axis parameter, the most noticeable change in the electronic structures takes place in the d_{xy} band: the d_{xy} band shifts up in energy around **Γ** and shifts down around **M**, while d_{xz}/d_{yz} bands exhibit little change (Fig. S13). Phenomenologically, as the diagonal hopping between d_{xy} orbitals is sensitive to Se height while d_{xz}/d_{yz} is not, a small variation in Se height will mainly affect the d_{xy} bands. With a decrease in *a*-axis, the Se height increases and this leads to a reduction of the coupling between Fe d_{xy} and Se p_x/p_y orbitals. Because the effective hopping between d_{xy} orbitals from the above coupling is negative, this reduction is expected to increase the diagonal hopping between d_{xy} orbitals, which, in turn, causes the d_{xy} band's upshift around **Γ** and downshift around **M**.

It should be noted that all DFT calculations are done for the tetragonal phase (at high temperatures, i.e. $T > T^*$), where the d_{xy} electron Fermi pocket shows only slight changes, consistent with the overlapped Hall coefficient at high temperatures. The experimentally-observed dramatic change of the electron density occurs at low temperatures.

Due to the fact that the nematic phase and other strong correlation effects at low temperatures cannot be fully captured in DFT, the absolute number of the change in the electron density cannot be extracted from our calculations. Nevertheless, the most important piece of information from the calculations here is that the band dispersion of the d_{xy} orbital is more sensitive to the lattice change than the $d_{xz}/_{yz}$ bands. The shift of the d_{xy} orbital bands will have a significant effect on the corresponding band splitting at low temperatures (nematic phase), which may lead to a more dramatic change in the electron density.

References

1. D. S. Ivanov, L. V. Zhigilei, Effect of Pressure Relaxation on the Mechanisms of Short-Pulse Laser Melting. *Phys. Rev. Lett.* **91**, 105701 (2003).
2. T. E. Itina, J. Hermann, P. Delaporte, M. Sentis, Laser-generated plasma plume expansion: Combined continuous-microscopic modeling. *Phys. Rev. E* **66**, 066406 (2002).
3. H. Hu, *et al.*, Quantum Electronic Stress: Density-Functional-Theory Formulation and Physical Manifestation. *Phys. Rev. Lett.* **109**, 055501 (2012).
4. Z. Chen, *et al.*, General Synthesis of Dual Carbon-Confined Metal Sulfides Quantum Dots Toward High-Performance Anodes for Sodium-Ion Batteries. *Adv. Funct. Mater.* **27**, 1702046 (2017).
5. G. Kresse, J. Furthmüller, Efficiency of ab-initio total energy calculations for metals and semiconductors using a plane-wave basis set. *Comput. Mater. Sci.* **6**, 15 (1996).
6. W. Kohn, L. J. Sham, Self-Consistent Equations Including Exchange and Correlation Effects. *Phys. Rev.* **140**, A1133 (1965).
7. J. P. Perdew, *et al.*, Atoms, molecules, solids, and surfaces: Applications of the generalized gradient approximation for exchange and correlation. *Phys. Rev. B* **46**, 6671 (1992).
8. J. P. Perdew, K. Burke, M. Ernzerhof, Generalized Gradient Approximation Made Simple. *Phys. Rev. Lett.* **77**, 3865 (1996).
9. S. Grimme, Semiempirical GGA-type density functional constructed with a long-range dispersion correction. *J. Comput. Chem.* **27**, 1787 (2006).
10. H. J. Monkhorst, J. D. Pack, Special points for Brillouin-zone integrations. *Phys. Rev. B* **13**, 5188 (1976).
